# Numerical simulation of stress wave interaction in short-delay blasting with a single free surface

**DOI:** 10.1371/journal.pone.0204166

**Published:** 2018-09-26

**Authors:** Xianyang Qiu, Yifei Hao, Xiuzhi Shi, Hong Hao, Shu Zhang, Yonggang Gou

**Affiliations:** 1 School of Resources and Safety Engineering, Central South University, Changsha, People’s Republic of China; 2 Department of Civil Engineering, School of Civil and Mechanical Engineering, Curtin University, Bentley, WA, Australia; 3 Key Laboratory of Coast Civil and Structure Safety of Ministry of Education, Tianjin University, Tianjin, People’s Republic of China; Politecnico di Milano, ITALY

## Abstract

It is generally believed that stress wave superposition does occur and plays an important role in cutting blasting with a single free surface, in which explosive columns of several blast holes with short spacing are simultaneously initiated. However, considering the large scatter of pyrotechnic delay detonators that are used in most underground metal mines in China, the existence of stress wave superposition and the influence of this effect on rock fragmentation are questionable. In the present study, the stress wave interaction in short-delay blasting with a single free surface was studied through the use of the LS-DYNA code. Stress waves induced by two blast holes blasting with different delays were compared with the single blast hole case, and the effects of delay time, detonating location and spacing on stress wave superposition were investigated. The numerical results showed that for blast holes with a 1 m spacing, stress wave interaction only occurs when the delay time is 0 ms and does not occur for blasting with delays of more than 1 ms. An increase in the duration of a stress wave via optimizing the detonation location does not improve the stress wave interaction. For a 1 ms delay, stress wave superposition only occurs when the spacing is more than 4 m, which is a rare case in practice. The results indicated that the occurrence of stress wave superposition for blasting with a single free surface is strictly limited to conditions that would be difficult to achieve under the existing delay accuracy of detonators. Therefore, it is unrealistic to improve fragmentation via the stress wave interaction in field blasting. Furthermore, the numerical results of the stress wave interaction also show that there would be a great potential to reduce the hazardous vibrations induced by short-delay blasting by using electronic detonators with better control of delays in an order of several milliseconds.

## Introduction

Delay blasting has been proved, by both practical engineering and experimental tests, to result in better fragmentation and less vibration than simultaneous blasting [1, 2]. Over the years electronic detonators, which have an accuracy of normally 1 ms, have been one of the biggest breakthroughs in blasting technology. An improvement of fragmentation has been seen in many mines after using electronic detonators [3]. Researchers and blasting practitioners had, through many field experiments and trails, arrived at the consensus that precise delay blasting using electronic detonators had significant advantages as regards the improvement of rock fragmentation [4–6].

The reason for the improvement of fragmentation in rock blasting with the use of electronic detonators is still under discussion. A widely recognized reason is that the reduced initiating scatter results in no hole firing out of planned order [7]. Another controversial reason is that since short-delay blasting is possible with the increase of initiating accuracy for detonators, stress waves interaction between adjacent blast holes does occur and plays an important role in rock fragmentation [8–10]. Rossmanith [9] and Rossmanith and Kouzniak [10] constructed a 2D model to study the possible area of shock wave interaction and showed how a positive effect of shock wave interaction could be achieved in an charge column with infinitely length. Chiappetta [11] indicated that short-delay internals such as 2 ms, which was short enough for stress waves from adjacent blast holes to interact with each other, was beneficial to improve fragmentation. McKinstry [12] suggested a delay time of 3 ms for the application of electronic detonators at Barrick and argued that the delay time should be selected to create stress wave superposition. In contrast, plenty of field trails and experimental tests haven been done to investigate the influence of delay times on rock fragmentation[13–16]. Although different delay internals for optimum fragmentation were obtained, these tests indicated that the delay times which produced best fragmentation were much longer than those which created stress wave collision. Blair [17] extensively discussed the limitations of most assumptions which supported the hypothesis that the use of electronic detonators improved fragmentation, and concluded that the role of stress wave interaction in rock fragmentation was unpredictable and unimportant.

Some studies were conducted to compare the blasting performance of rock fragmentation between delays which created stress wave interaction and those that avoided the interaction. Vanbrabant and Espinosa [18] argued that the delays should be selected to create an overlap of the P-wave particle velocity and their field observations showed a nearly 50% increase in the average fragmentation. In contrast, small-scale tests performed by Johansson and Ouchterlony [19] indicated that the rock fragmentations with delays in the time range of shock wave interaction were statistically same with delays out of shock wave interaction. Yi et al [20] constructed a four-hole model to study the impact of short delays on rock fragmentation and indicated that increased fragmentation was found for longer delays (3 ms and 6 ms) compared with simultaneous initiation and 1ms delay. Similar simulations in sublevel caving by Yi et al [21] showed that the 2 ms case gave a finer fragmentation than the 0 ms and 1 ms cases. The existence of the stress wave superposition in short delay blasting was also questioned by some researchers. The numerical study of Schill and Sjöberg [22] indicated that the effect of stress wave interaction was local around the interaction plane, which implied that the short delay blasting did not result in improved fragmentation. Yi et al. [23] carried out both analytical and numerical studies to investigate the stress wave superposition between two adjacent holes and their results also did not support the hypothesis that the stress interaction could improve fragmentation.

**Fig 1:**
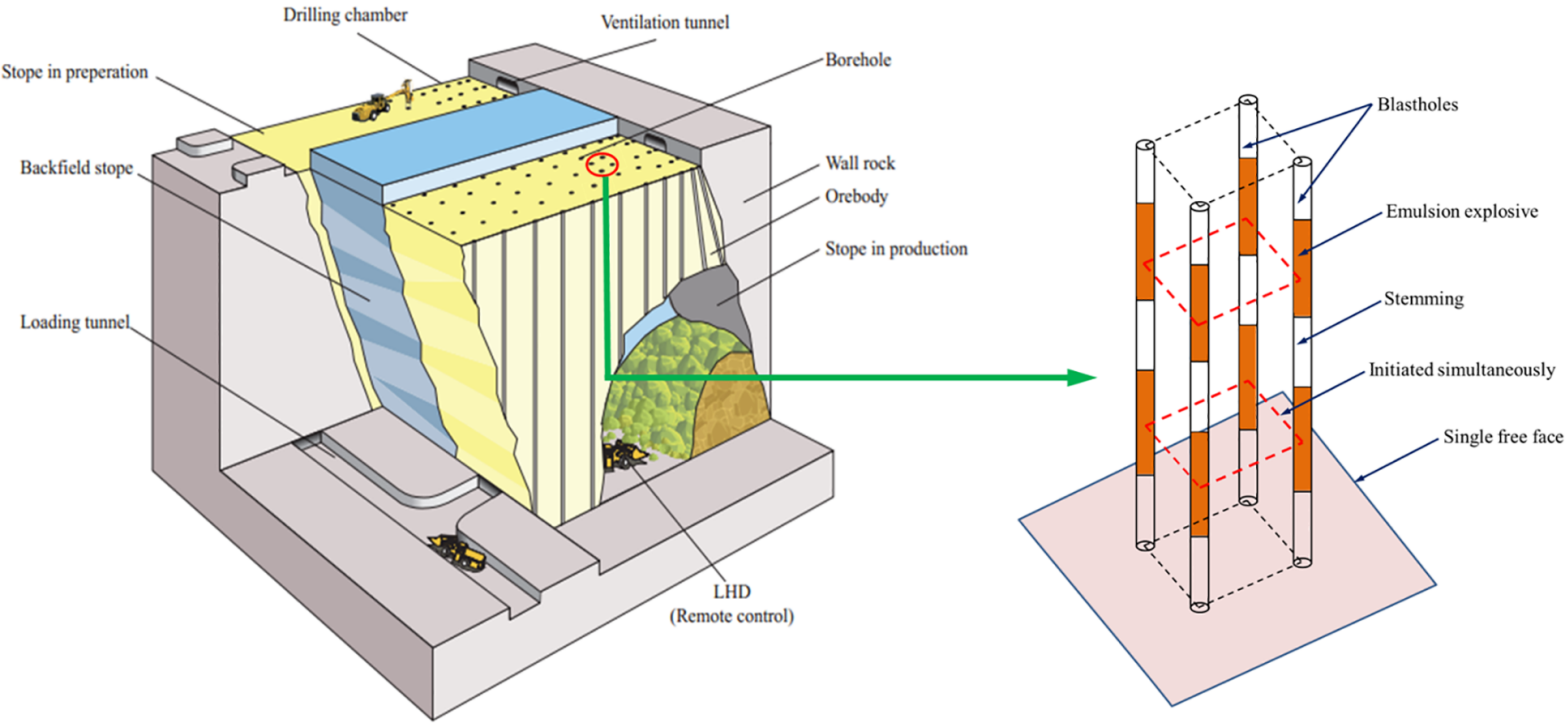
Cutting blasting with a single free surface in underground mines.

**Fig 2:**
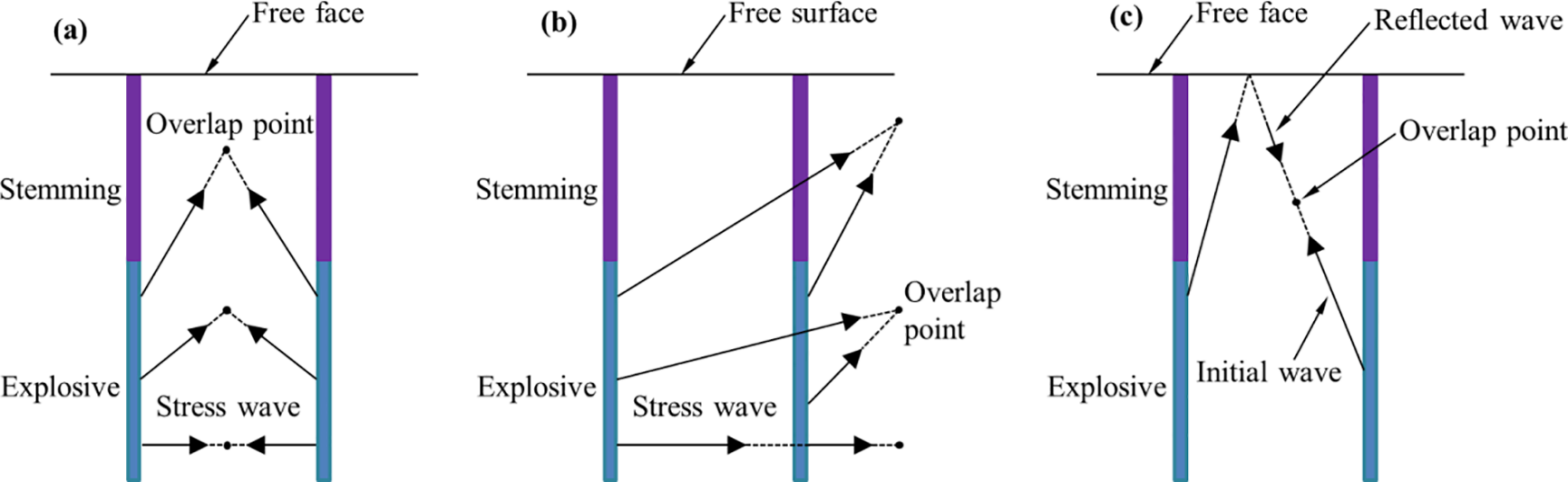
Interaction of stress waves between two adjacent blast holes with short delay. (a) interaction of initial waves within the range of holes; (b) interaction of initial waves outside the range of holes; (c) interaction of initial wave and reflected wave.

Previous studies regarding short delay blasting were mostly conducted in opencast mines. Blasting in underground engineering often associates with the situation of only a single free surface, which is different from open-pit engineering [24]. In order to artificially create new free surfaces for subsequent blasts, there can be a set of blast-holes close in space and initiating at the same nominal delay time (a typical cutting blasting scheme in underground mines is shown in Fig 1), which results in large charge weight per delay [25]. The short delay blasting with single free surface was proposed by Qiu et al. [26] to reduce the harmful vibration in underground cutting blasting. The objective of this study is to investigate the effect of stress wave interaction between blast holes with a single free surface. Compared to the 2D model with the assumption of an infinitely long charge length presented in the previous studies of Rossmanith [9] and Yi et al. [23], the stress wave interaction in a 3D model of short-delay blasting with a single free surface is much more complicated. As seen in Fig 2, the existence of a free surface would lead to additional superposition of the reflected stress wave from the first blast hole with the incoming stress wave from the second blast hole (Fig 2C), which affects the rock fragmentation process. If the effect of stress wave interaction does occur and plays an important role in rock fragmentation, the delay times in underground blasting should be selected to make use of this superposition effect for better fragmentation by using electronic detonators. If the stress wave interaction is not indispensable in rock blasting, the delay times in short delay cutting blasting can be much longer, which will be of great benefit to reduce the hazardous vibrations induced by cutting blasting with single free surface.

In this study, 3D models of one blast hole and two blast holes with a free surface are established in LS-DYNA code. The stress wave from blast in a single blast hole is simulated and compared with that from two adjacent blast holes with or without delays. The influence of delay time, detonating location and spacing between blast holes on the effect of stress wave superposition is investigated. It should be noted that in this study, compressive stress is negative and tensile stress is positive.

## Numerical model

In the present study, LS-DYNA [27] was used for the numerical simulation. Its accuracy in simulating the response of rock mass under blast loading has been proven in previous studies [28–31].

### FE model

**Fig 3:**
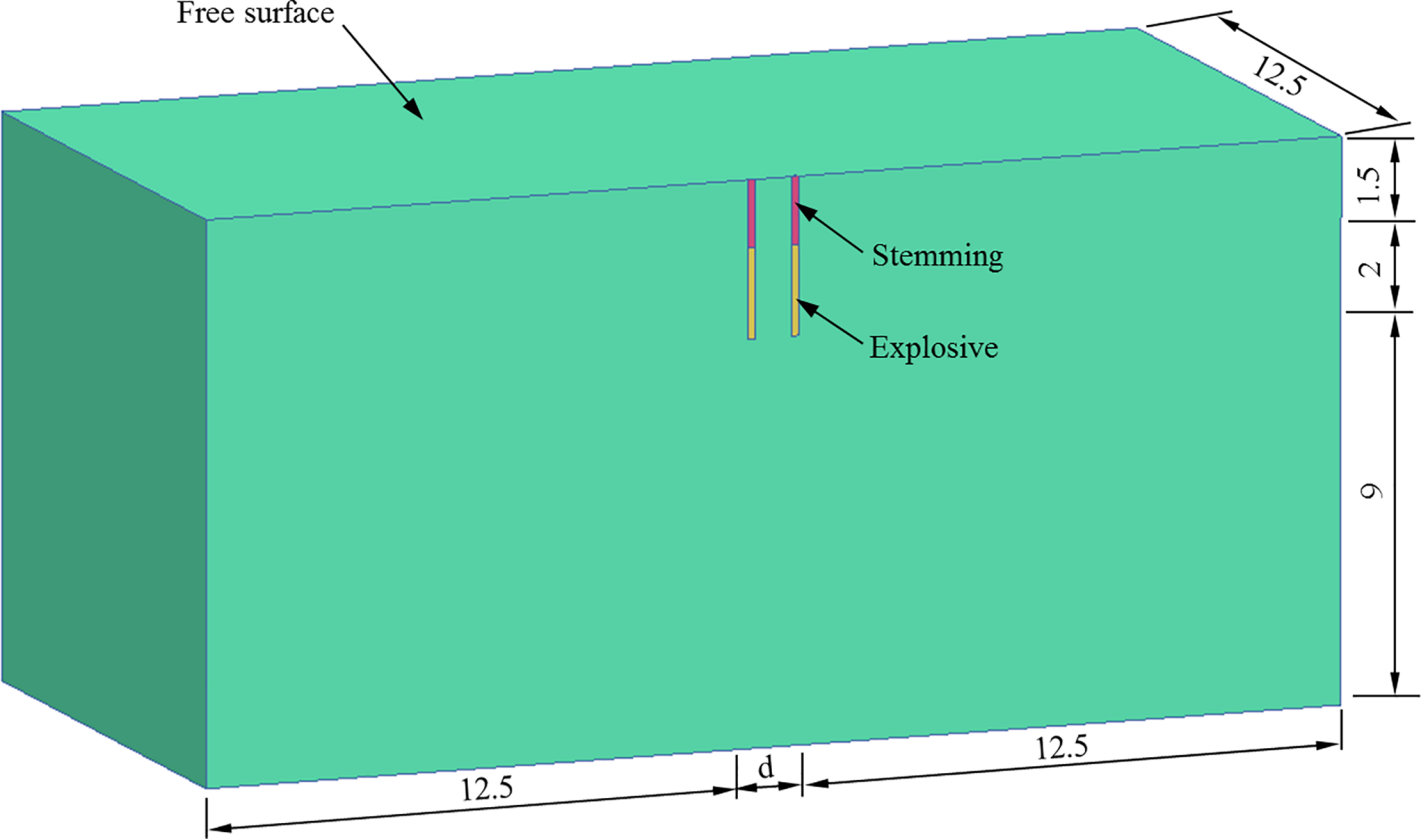
Geometry of model with two blast holes.

Fig 3 shows the 3D model considered in this study. The simulation begins with a single blast hole model, and then two blast holes with different separations are simulated. The blasting parameters are selected according to the practical cutting blasting in underground mines. To reduce the computational time, only half of the considered range is included in the numerical models by assuming symmetry. The blast holes, with a diameter of 160 mm, are perpendicular to the free surface. The length of the blast holes is 3.5 m, in which 2.0 m is the explosive column and 1.5 m is stemming near the free surface. To eliminate the influence of the model size on the analysis results due to wave reflections from numerical boundaries, single blast hole models with different dimensions were simulated first, and the dimensions are finally determined as 25 m×12.5 m×12.5 m (length×width×height) for the model with a single blast hole and 12.5 m×12.5 m (width×height) with different lengths (25+d m) for the models with two blast holes, in which d is the separation between the two holes, as illustrated in Fig 3. The results, which are not shown here, indicate the reflected waves with these model sizes from the numerical boundary do not affect the simulation results in this study.

**Fig 4:**
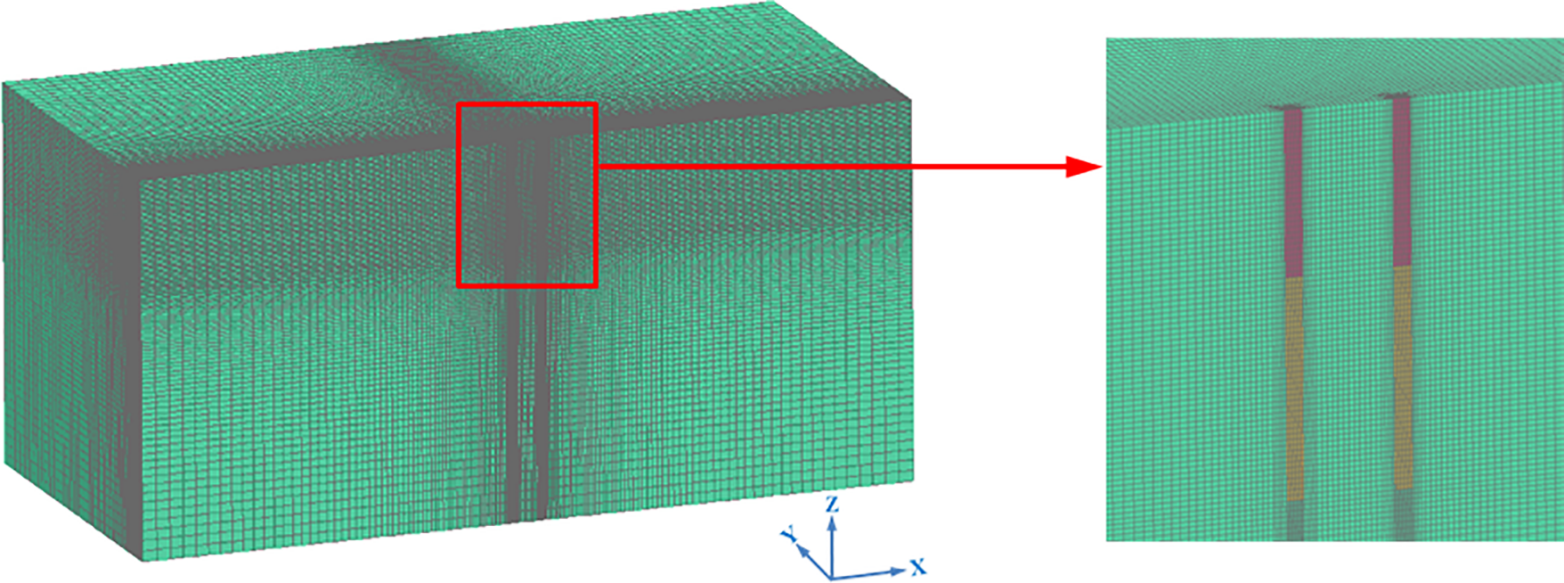
FEM mesh for model with two blast holes with 1 m spacing.

In this simulation, the arbitrary Lagrangian–Eulerian (ALE) method, which allows a mesh to move independent of the material flow, is used to model the rock blasting [32]. The model is divided into a mesh of hexahedron-shaped brick elements with eight nodes. Suggested by Kuhlemeyer and Lysmer [33], the mesh size should be shorter than 1/8–1/10 of the wavelength in general to reduce any wave distortion. Convergence tests are conducted to determine the sizes of elements until the difference of the modelling results between two consecutive element sizes is less than 5%. Based on the convergence test results, which are not shown here, the element size in the numerical model varies from 50 mm near the explosive to 200 mm close to the boundary. The FEM mesh for the model with two blast holes with 1 m spacing is shown in Fig 4, which has a total of 1458860 elements and 1502570 nodes. The boundary conditions are specified as follows: the top boundary is the free surface, the front boundary is the symmetric boundary, and the others are non-reflecting boundaries.

### Rock mass

Rock in the zone close to the charge holes will rapidly enter a state of high pressure and large strain after explosives detonate. Therefore, the elastic-plastic dynamic failure constitutive model with kinematic hardening is adopted in the present study to model rock responses under high explosive pressure. For this case, the Cowper and Symonds model, which can describe the strain rate effect and plastic hardening of rock, is employed in the analysis [34]:

$$\sigma_{y}=\left[1+{\left(\frac{\dot{\varepsilon }}{C}\right)}^{1/P}\right]\;(\sigma_{0}+\beta E_{P}\varepsilon_{P}^{eff})$$ (1)

$$E_{P}=\frac{EE_{tan}}{E-E_{tan}}$$ (2)

where *P* and *C* are the user-defined input constants, valued as 4.0 and 2.5 s^-1^, respectively; $\dot{\varepsilon }$ is the strain rate; *σ*_*y*_ and *σ*_0_ are the current and initial yield strength, respectively. The parameter *β* ranges from 0 to 1, denoting kinematic hardening and isotropic hardening, respectively. The parameters *E*_*P*_, *E* and *E*_*tan*_ are the plastic hardening modulus, Young’s modulus and tangential modulus, respectively. $\varepsilon_{P}^{eff}$ is the effective plastic strain, derived from:

$$\varepsilon_{P}^{eff}=\int_{0}^{t}{\left(\frac{2}{3}{\dot{\varepsilon }}_{ij}^{P}{\dot{\varepsilon }}_{ij}^{P}\right)}^{1/2}dt$$ (3)

where *t* is the accumulative time after plastic strain initiation and ${\dot{\varepsilon }}_{ij}^{P}$ represents the plastic deviatoric strain rate. Because the strain rates of rock material near the charge hole may range from 10^1^ to 10^5^ s^-1^, the strain rate dependence of the compressive strength and tensile strength for rock should be considered. Based on Li [35], the dynamic compressive and tensile strengths in the strain rate ranges can be estimated as:

$$\left\{ \begin{array}{c} \sigma_{cd}=\sigma_{c}{\dot{\varepsilon }}^{1/3}\\ \sigma_{td}=\sigma_{st}{\dot{\varepsilon }}^{1/3} \end{array}\right.$$ (4)

where *σ*_*cd*_ and *σ*_*td*_ are the dynamic compressive and tensile strengths of rock (Pa), respectively, and *σ*_*c*_ and *σ*_*st*_ are the static compressive and tensile strengths of rock, respectively.

The material properties for hard rock, which is the most typical rock in underground metal mines, are listed in Table 1. According to the relationship between the P-wave velocity, elastic modulus, density and Poisson’s ratio, the P-wave velocity is Cp = 4310 m/s in the rock mass.

**Table 1:** Parameters of rock mass.

*ρ* (kg/m^3^)	*E* (GPa)	*v*	*σ*_*c*_ (MPa)	*σ*_*st*_ (MPa)	*σ*_0_ (MPa)
2700	40	0.19	153	9.5	75

### Explosive

High explosives are modelled by the John-Wilkins-Lee (JWL) equation, which is the most commonly used equation for modelling high explosives due to its simple form, experimental basis, and easy calculations of hydromechanics [27]. The JWL equation is given as follows:

$$P=A\;\left(1-\frac{\omega }{R_{1}V}\right)\;e^{-R_{1}V}+B\;\left(1-\frac{\omega }{R_{2}V}\right)\;e^{-R_{2}V}+\frac{\omega E}{V}$$ (5)

where P is the pressure (dependent variable); *A*, *B*, *R*_1_, *R*_2_, and *ω* are constants; *V* is the specific volume; *E* is the specific internal energy. The #2 rock emulsion explosive is most commonly used in underground metal mines; thus, the material parameters for the explosive in the present study are as follows: A = 220 GPa, *B* = 0.2 GPa, *R*_1_ = 4.5, *R*_2_ = 1.1, *ω* = 0.35, and *E* = 4.2 GPa. The detonation velocity *D* and mass density of the emulsion explosive *ρ*_0_ are 3600 m/s and 1000 kg/m^3^, respectively.

### Stemming

Material Type 5 of the LS-DYNA (*MAT_SOIL_AND_FOAM) is used for the stemming. The parameters of stemming in the simulation are listed in Table 2 [36].

**Table 2:** Parameters of stemming.

*ρ* (kg/m^3^)	*v*	*E*_*T*_ (GPa)	*Cohesive force* (MPa)	μ	φ (°)
1800	0.35	16	0.018	9.5	35

## Simulation results of single blast hole blasting

### Attenuation relation of blasting stress wave

In rock blasting, it is generally agreed that with the detonation of the explosive columns in boreholes, shock waves are generated and propagate in the rock mass and attenuate after travelling a certain distance [37]. The attenuation characteristics of shock waves (stress waves) induced by rock blasting with distance can be described by [38, 39]:

$$P_{r}=P_{d}\;{\left(\frac{d}{r}\right)}^{\alpha }$$ (6)

$$P_{d}=\frac{\rho_{0}D^{2}}{1+\gamma }\frac{2\rho C_{P}}{\rho C_{P}+\rho_{0}D}$$ (7)

where *P*_*r*_ is the peak pressure of shock/stress waves at a distance r from the explosive source in a rock mass, *P*_*d*_ is the pressure on the rock interface in the blasting hole, *d* is the diameter of the charge, and *α* is the attenuation coefficient. In the propagation area of a shock wave, $\alpha =2+\frac{v}{1+v}$; in the propagation area of a stress wave, $\alpha =2-\frac{v}{1+v}$; *v* is Poisson’s ratio of the rock mass; ρ is the density of the rock mass; ρ_0_ is the density of the explosive; D is the detonation velocity of the explosive; γ is the adiabatic parameter of detonation products; C_P_ is P-wave velocity of the rock mass.

**Fig 5:**
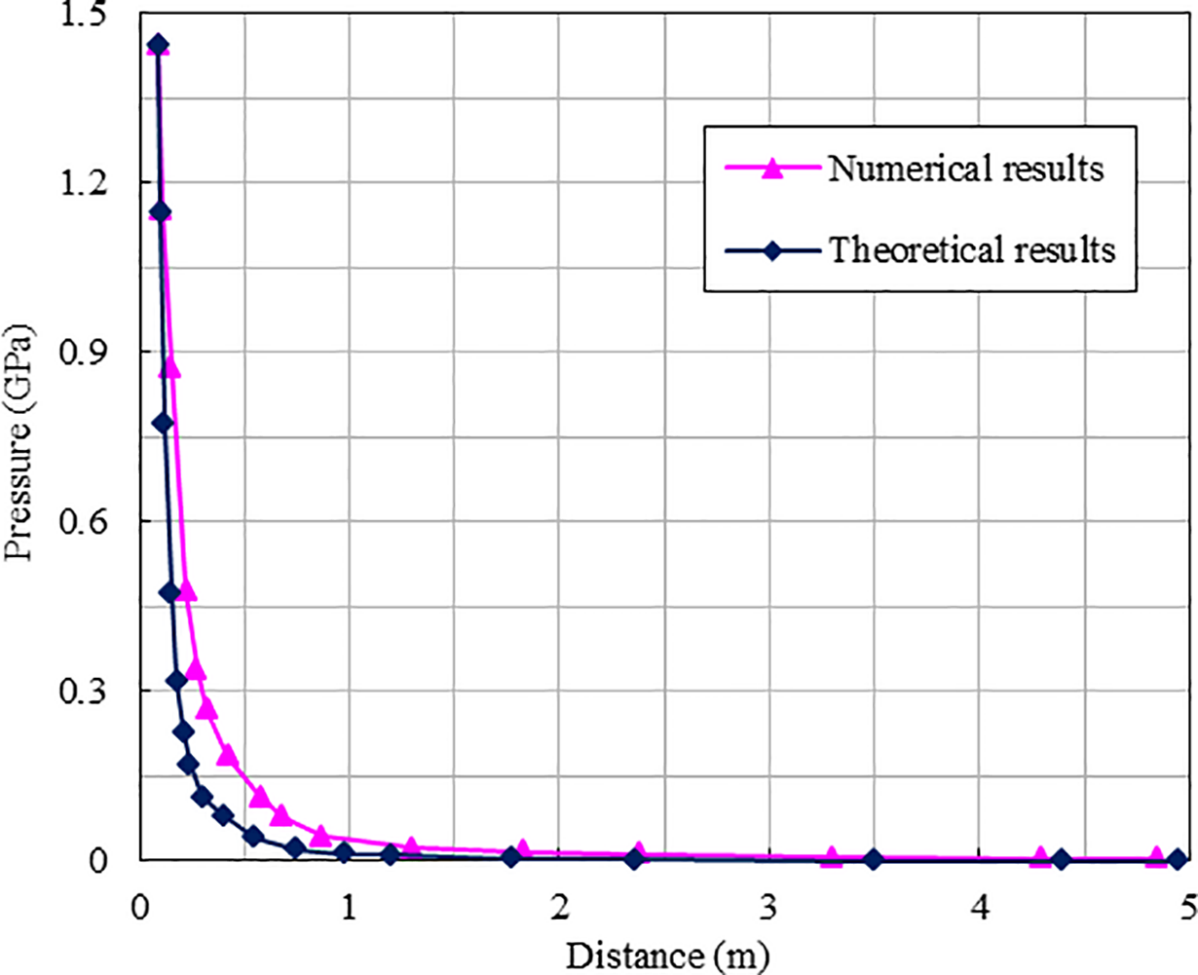
Comparison of peak pressures attenuation of rock mass.

Single hole blasting was simulated to verify the numerical model. Fig 5 shows a comparison of the theoretical and numerical attenuation curves of peak pressures, along with distances from the centre of the explosive. It can be seen that the attenuation curve obtained by the numerical simulation agrees very well with the results of the theoretical calculation. This demonstrates the reliability of the numerical model in simulating blasting wave propagation in rock mass. In addition, from Fig 5, we can clearly see the attenuation characteristic of the stress wave induced by blasting in the rock mass as follows: the peak pressure rapidly attenuates from nearly 1.5 GPa near the charge column to less than 100 MPa at a distance of 1.0 m from the blast hole.

### Radial stress

To further verify the numerical model, the simulated radial stress from a single hole blasting is compared with the recorded data in a blasting test [32]. The explosive used in the test was Iregel 1175U, with a density 1250.0 kg/m^3^ and detonation velocity 6178 m/s. The explosive column, with a length of 2.5 m and a radius of 0.081 m, was centred 3.75 m below the ground surface and had the bottom detonation type. The rock mass was oil shale, with a density of 2261 kg/m^3^. Details of the rock mass and explosive were given by Wang et al [32]. These parameters are adopted in the present numerical model to simulate the test.

**Fig 6:**
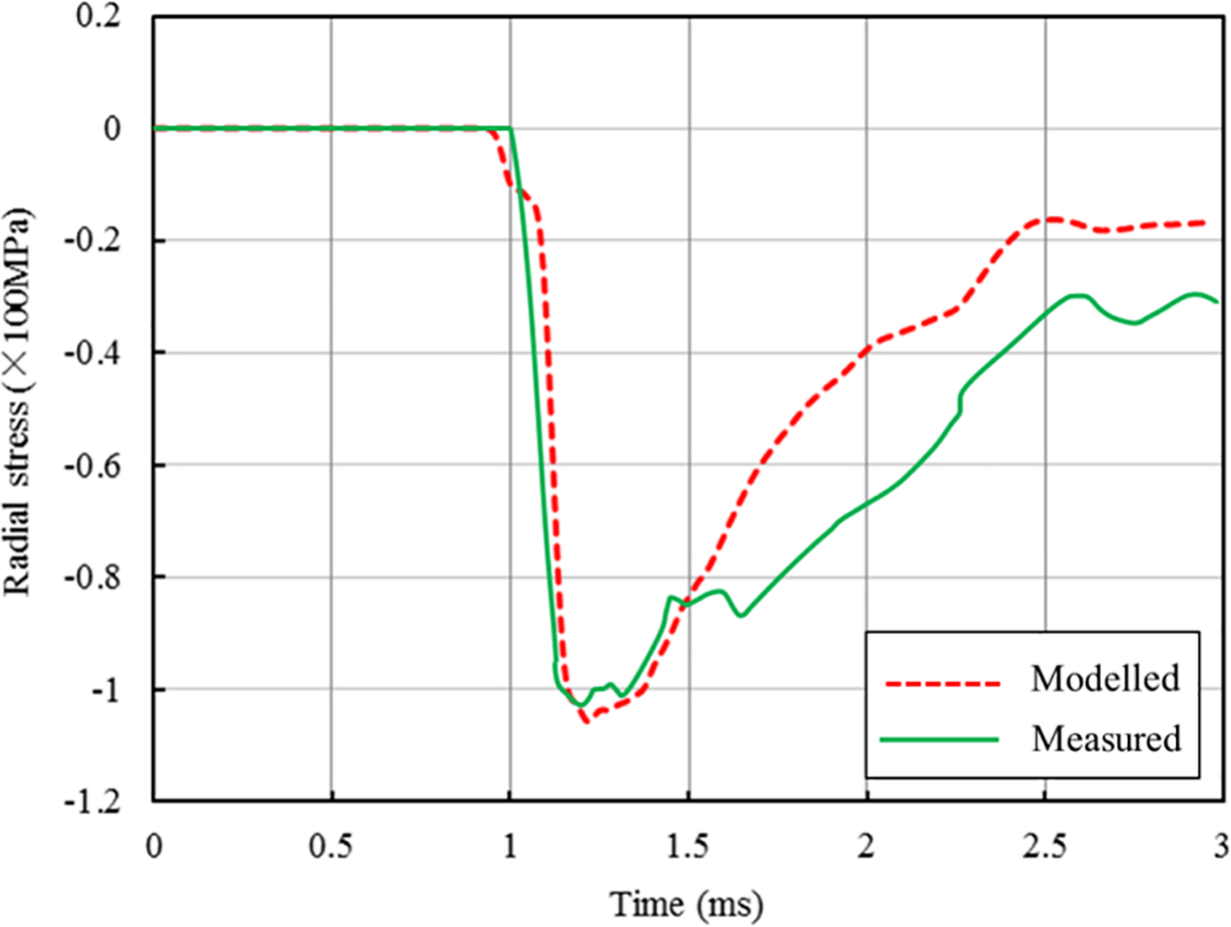
Comparison of radial stresses between numerical and recorded data.

Fig 6 shows the comparison of the modelled and recorded radial stresses at a point 2.5 m below the free face and 3.0 m away from the explosive centre. As shown, the rapid increment after stress wave arrival and the peak value obtained from the numerical simulation agree well with the test data. In addition, a similar attenuation trend can be found in the stress declining stage of the two radial stress wave curves. The smaller values of the simulated radial stress compared with the recorded data in the declining stage are due to the complexity of rock geology in blasting experiments. This comparison demonstrate again the numerical model can give reasonable predictions of blast wave propagations in rock mass.

## Analysis of stress wave interaction of short-delay blasting

**Fig 7:**
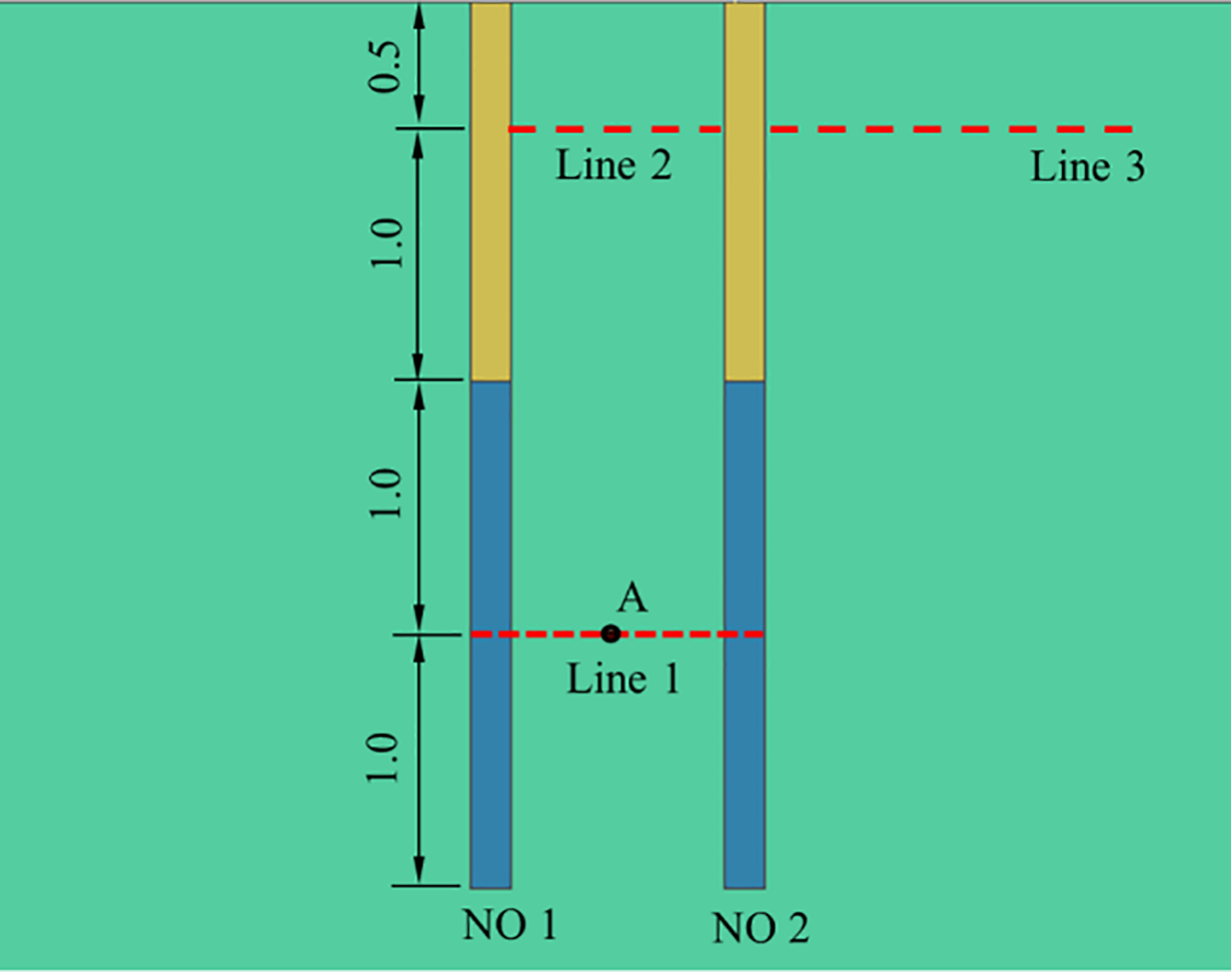
Monitoring scheme.

Three parameters, including the delay time, detonating location and spacing between blast holes, which might affect the blast wave interaction between two adjacent blast holes, are considered herein. As the accuracy of an electronic detonator is ±1 ms, it is impossible to reliably implement blasting with a delay time less than 1 ms in practice. Therefore, apart from the 0 ms case, the unit of delay times studied in the present study is 1 ms. For crater blasting with two blast holes, the stress waves within the range of the blast holes in the charging segment are of the primary interests because of its intensity for breaking the rock. Apart from those, stress waves near the free face, both in between and outside the range of the blast holes are also considered to form a larger blasting crater. Fig 7 shows the monitoring scheme in the present study. In the charging segment, the stress waves between two blast holes are measured at line 1. Near the free surface, the stress waves are measured at line 2 between blast holes and at line 3 on the right side of the second blast-hole NO.2. Both lines 2 and 3 are 0.5 m from the free surface.

### Effect of delay time on stress wave interaction

**Fig 8:**
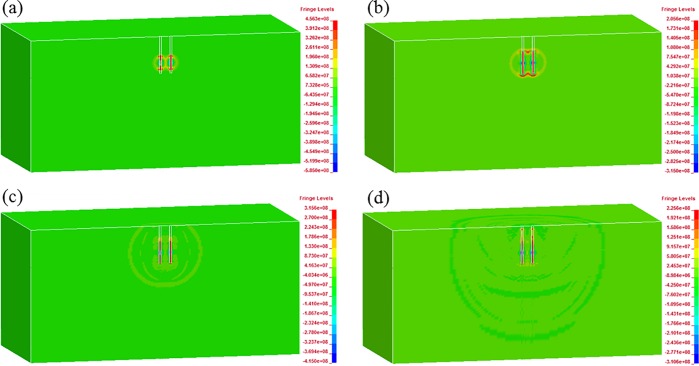
Blast wave propagation and its interaction between simultaneously initiated holes with 1 m spacing: (a) 0.20 ms; (b) 0.36 ms; (c) 0.80 ms; (d) 2.30 ms.

**Fig 9:**
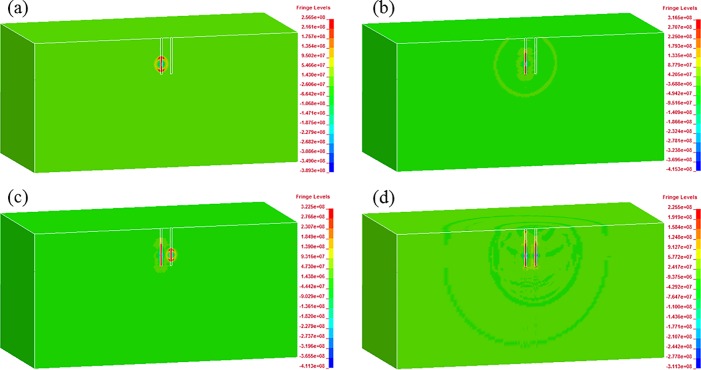
Blast wave propagation and its interaction between 1 ms delayed holes with 1 m spacing: (a) 0.24 ms; (b) 0.36 ms; (c) 1.15 ms; (d) 2.30 ms.

In order to study the blast wave propagation and its interaction, Figs 8 and 9 show the blast pressure for two adjacent blast holes with 1 m spacing when the delay times are 0 ms (simultaneous initiation) and 1 ms, respectively. In Fig 8, we can clearly see the stress wave superposition between those from simultaneously detonated holes, especially within the range of the two holes. For the 1 ms delay case shown in Fig 9, no such superposition phenomenon can be found both within and outside the range of the blast holes. When the column of the second blast hole initiates, blasting waves induced by the first one have already passed far away owing to the very short blast wave duration.

**Fig 10:**
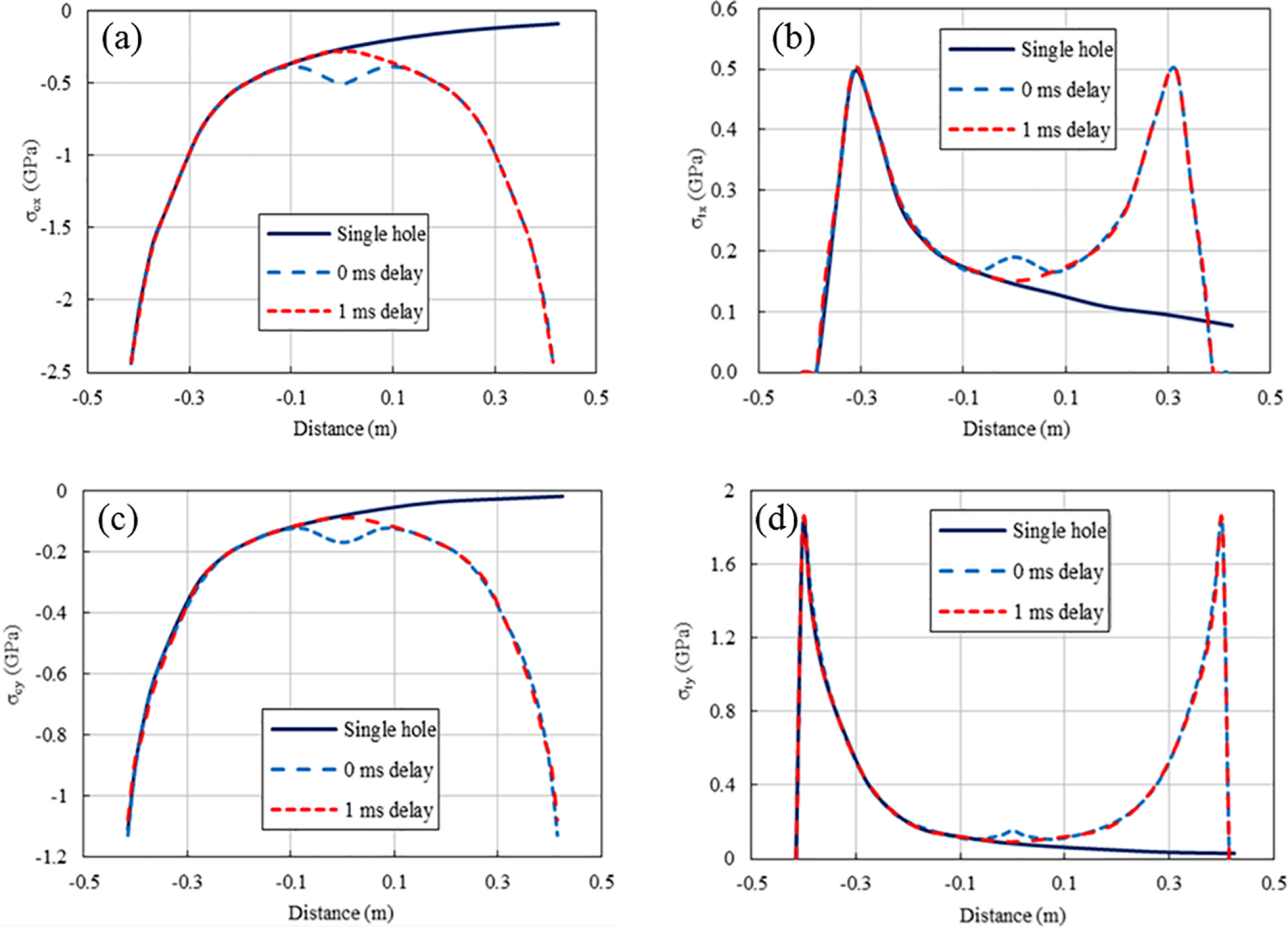
**Peak stresses of blasting in holes with 1 m spacing at line 1:** (a) Peak compressive stresses in X direction; (b) Peak tensile stresses in X direction; (c) Peak compressive stresses in Y direction; (d) Peak tensile stresses in Y direction.

**Fig 11:**
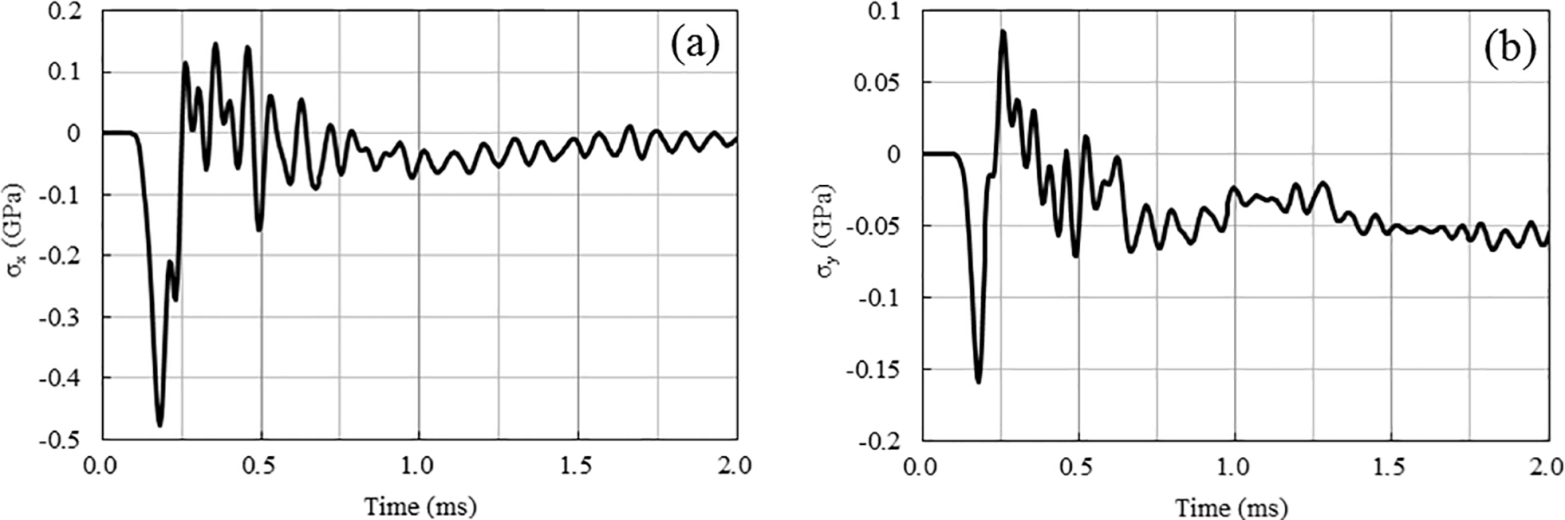
**Stresses for 0 ms delay case at point A:** (a) Stresses in X direction; (b) Stresses in Y direction.

**Fig 12:**
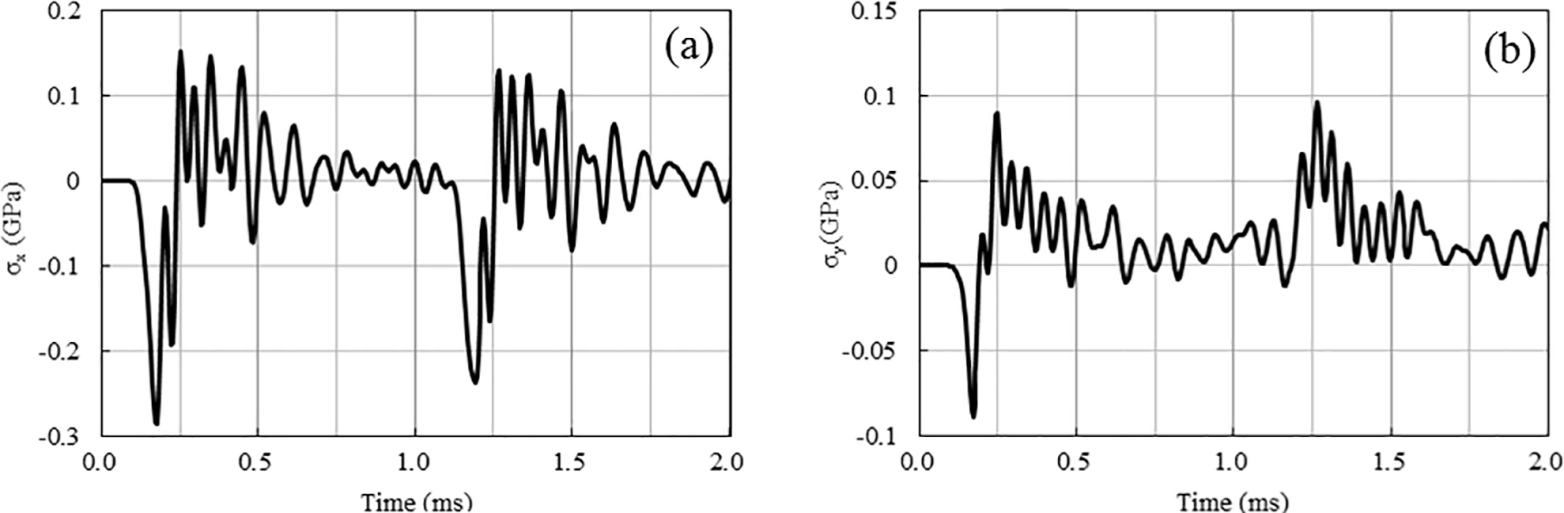
**Stresses for 1 ms delay case at point A:** (a) Stresses in X direction; (b) Stresses in Y direction.

Fig 10 shows the peak compressive and tensile stresses at line 1 for the single-hole blasting and two-hole blasting with 0 ms and 1 ms delays. As shown, the peak stress of the single-hole blasting attenuates very quickly. For instance, the peak compressive stress reduces from a peak of 2.43 GPa near the blast hole to less than 100 MPa within a distance less than 1.0 m away from the explosive centre, while the peak tensile stress reduces from approximately 500 MPa to less than 80 MPa. Compared to single-hole blasting, the peak stresses for the 0 ms delay cases experience an increase in the middle area between two blast holes, while such increase does not occur in the 1 ms delay case. For example, the peak compressive and tensile stresses for the 0 ms delay at the centre point between the two blast holes are approximately 500 MPa and 190 MPa, respectively. However, for the 1 ms delay case, they are approximately 285 MPa and 150 MPa, respectively, as in the single-hole case. In addition, as shown in Fig 10, the increase in peak stresses only occurs in a local area around the centre point of the two blast holes. This is due to the short duration of the blasting load and particle stress, which can be demonstrated by the time history curves of the stresses at point A (see Figs 11 and 12). From the numerical stress curves of the simultaneous detonation shown in Fig 11, apparent superposition of the stresses are found in both the X and Y directions. For the 1 ms delay case shown in Fig 12, the stresses induced by the explosive columns of different blast holes arrive at point A successively and separately. Therefore, stress wave superposition for blasting with a 1 m spacing only occurs in the 0 ms delay case within an extremely limited range.

**Fig 13:**
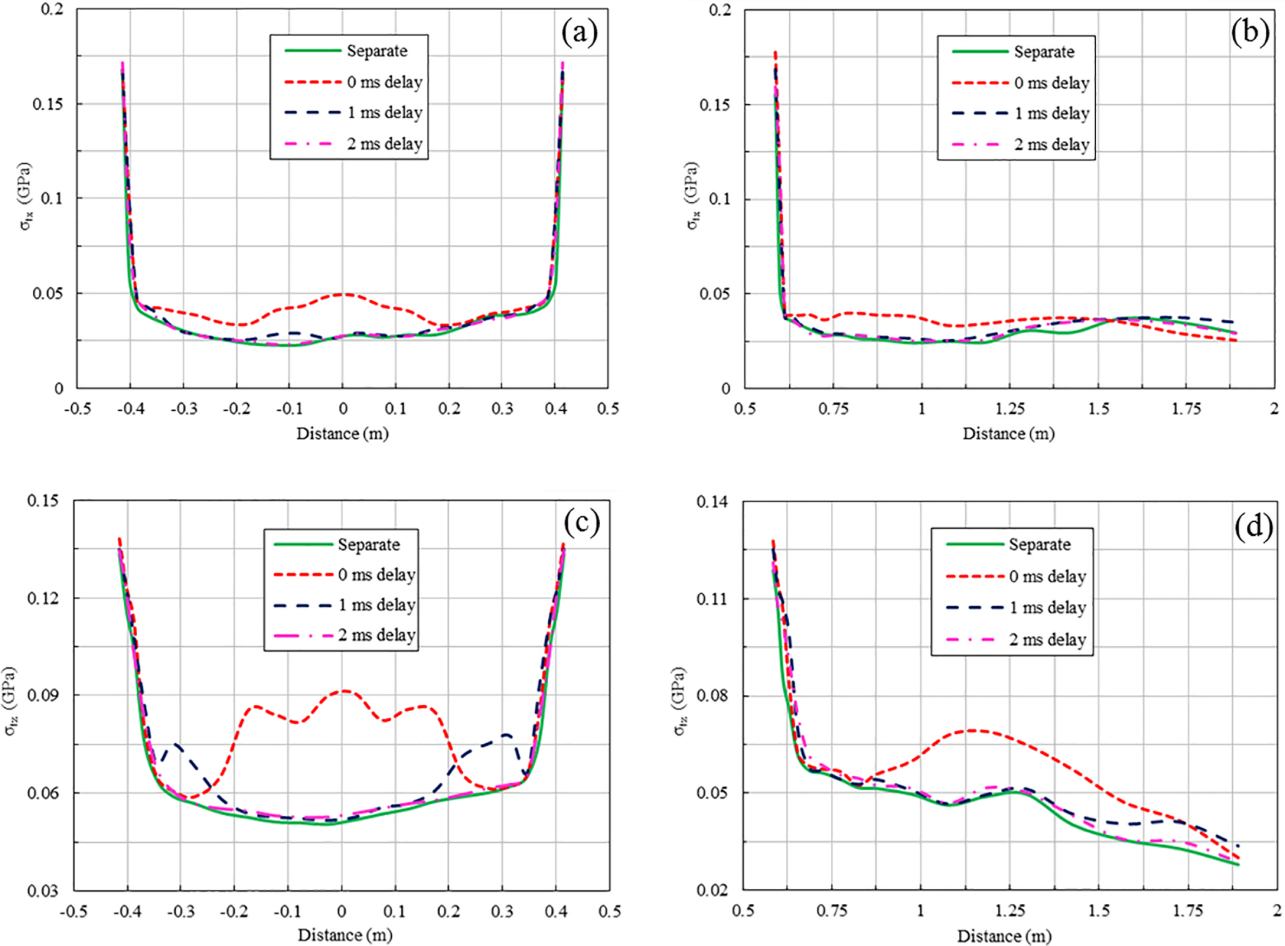
**Peak tensile stresses of 1 m-spacing blasting with different delays near free face:** (a) Peak tensile stresses in X direction along line 2; (b) Peak tensile stresses in X direction along line 3; (c) Peak tensile stresses in Z direction along line 2; (d) Peak tensile stresses in Z direction along line 3.

Fig 13 shows the peak tensile stresses of 1 m-spacing blasting with different delays near the free face. More number of simulations with different delays are considered and the results presented here, in which a case named ‘separate’ representing blasting in two holes with a 20 ms delay, i.e., interaction between stress waves from the two adjacent blast holes is very unlikely to occur. It can be seen from Fig 13 that distinct stress wave superposition can be found in the 0 ms delay case at both line 2 and line 3 compared with the ‘separate’ case. This is because the reflection of initial stress waves at the free surface leads to an increased duration of the stress waves. However, for the case of 1 ms delay, increase in the peak tensile stresses only occurs in local areas. Furthermore, the curves of the peak stress waves for the 2 ms delay case are almost the same as those in the separate case. This result indicates that although the stress waves near the free surface have longer duration, stress wave superposition does not occur for blasting with delays of more than 1 ms.

### Effect of detonating location on stress wave interaction

The duration of a stress wave plays an important role in the stress wave interaction. An increase in the duration of a stress wave is able to increase the scope and the possibility of wave superposition owing to interaction. Because of the limited detonation velocity of a cylindrical explosive, different detonation locations lead to different shapes and duration of the stress waves [40]. Therefore, the influence of detonating location on the stress wave interaction is examined in the present study. Three detonation locations are considered in this simulation: top detonation, centre detonation and bottom detonation.

**Fig 14:**
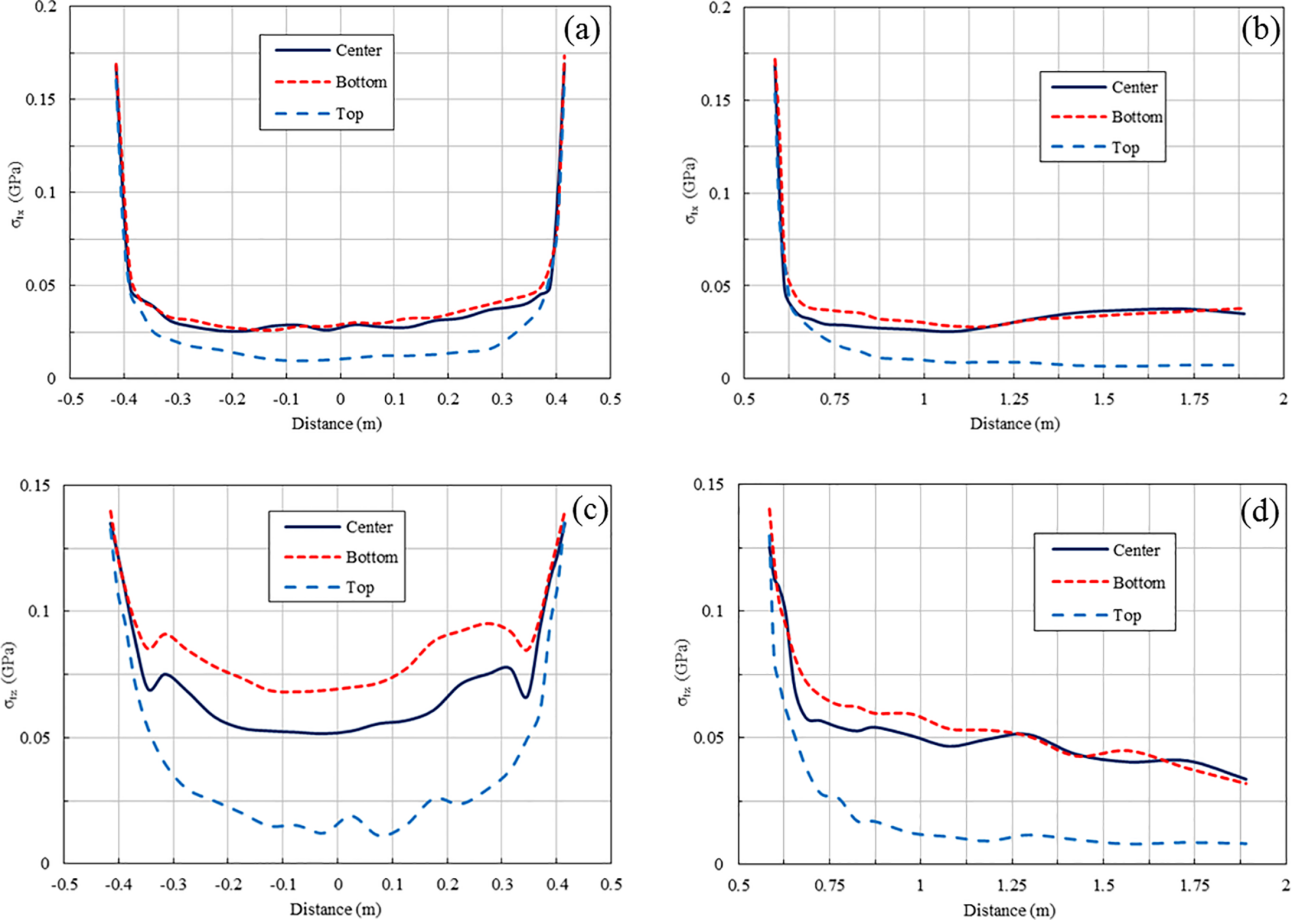
**Peak tensile stresses of 1 ms-delayed blasting for different types of explosive detonation near free face:** (a) Peak tensile stresses in X direction along line 2; (b) Peak tensile stresses in X direction along line 3; (c) Peak tensile stresses in Z direction along line 2; (d) Peak tensile stresses in Z direction along line 3.

**Fig 15:**
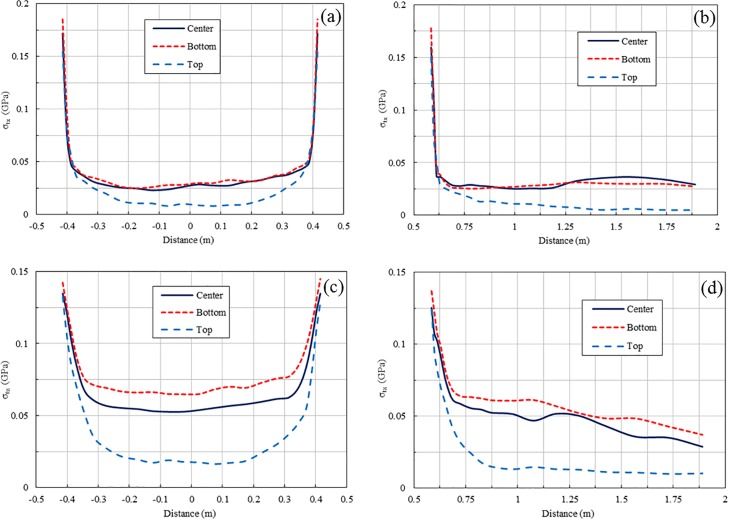
**Peak tensile stresses of 2 ms-delayed blasting for different types of explosive detonation near free face:** (a) Peak tensile stresses in X direction along line 2; (b) Peak tensile stresses in X direction along line 3; (c) Peak tensile stresses in Z direction along line 2; (d) Peak tensile stresses in Z direction along line 3.

The peak tensile stresses of 1 m-spacing blasting at different detonation locations for the 1 ms and 2 ms delay cases are presented in Figs 14 and 15, respectively. For the 1 ms delay case, an increase in the peak stresses occurs in several regions for all three types of detonation locations because of the stress wave superposition, while for the 2 ms delay case, stress wave superposition does not occur for all three detonation conditions. Obviously, the stress wave near the free surface induced by blasting for a top detonation has a longer duration than that for a bottom detonation. However, the peak tensile stresses of blasting for bottom and centre detonations are much larger than those for a top detonation in both the 1 ms and 2 ms delay cases. Therefore, the results from the numerical simulation indicate that an increase in duration of a stress wave via optimization of the detonation location does not improve stress wave interaction.

### Effect of spacing on stress wave interaction

**Fig 16:**
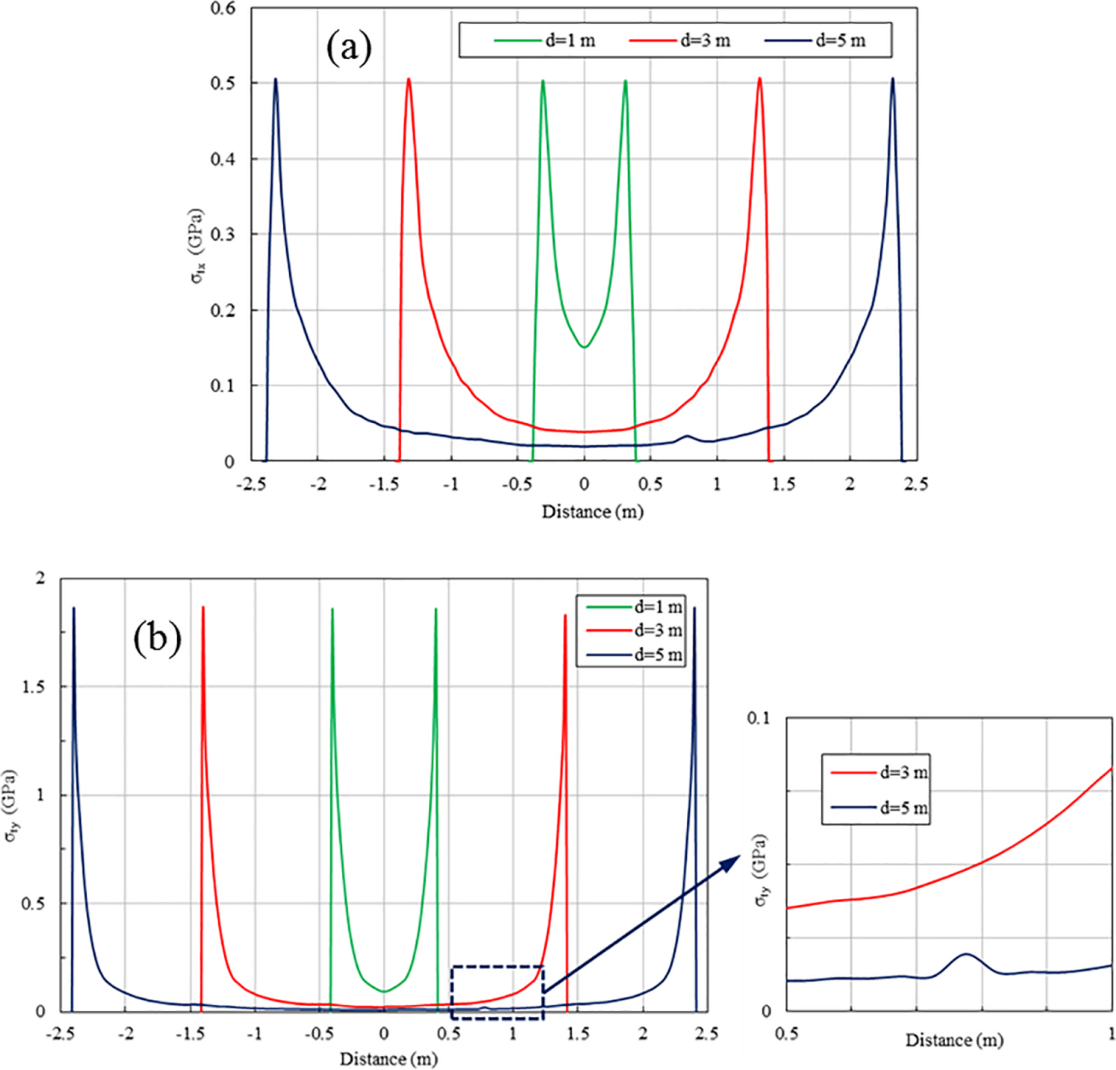
**Peak tensile stresses along line 1 in the range between two holes with for different separations:** (a) Stresses in X direction; (b) Stresses in Y direction.

With the increase in spacing, the stress wave induced by the first blast hole requires a longer time to propagate to the second blast hole and thus stress waves are more likely to undergo superposition. Two-hole blasting scenarios with different separation distances are simulated in the present study to investigate the effect of spacing on stress wave interaction. Fig 16 shows the peak tensile stresses for 1 ms-delayed blasting with different separations measured at line 1. Stress wave superposition does not occur in the cases of 1 m and 3 m separations, but it occurs around the point 0.8 m away from the centre for the 5 m spacing case. For a rock mass with a P-wave velocity of 4310 m/s (actually at locations very close to the blasting hole, blasting induced shock wave should propagate even faster), a 1 ms delay implies the blast wave from the first hole has travelled at least 4.31 m when blasting in the second hole takes place. Therefore for the case of 1 ms-delayed blasting in two holes with a spacing of less than 4.31 m, blasting wave interaction within the range of the two holes is very unlikely because the duration of blasting wave is very short and shock wave travels at a speed faster than the P-wave. Therefore, stress wave interaction only occurs when the spacing is more than 4.31 m for the 1 ms delay case. As shown for the case with a 5 m spacing, the superposition of the tensile stress in the X and Y directions occurs only in a very small range of 0.675–0.875 m and 0.725–0.825 m from the centre of the two holes, respectively. Furthermore, the peak tensile stress in the superposition area increases only from 27.0 MPa for single-hole blasting to 30.2 MPa in the X direction and from 13.3 MPa to 19.5 MPa in the Y direction, which is negligible for rock fragmentation. This result indicates that although stress wave superposition occurs as the spacing increases, the superposed stress wave is insufficient for rock breaking.

## Discussion and conclusions

For two adjacent blast holes with a 1 m spacing, no stress wave superposition occurs for a delay time between adjacent blast holes as low as 1 ms. Although a longer duration of stress waves can be obtained near the free surface, stress wave superposition only occurs in local areas for the 1 ms delay case and does not occur for blasting with delays of more than 1 ms. The numerical results also demonstrate that an increase in the duration of a stress wave via optimization of the detonation location does not improve the stress wave interaction. Hence, because the accuracy of an electronic detonator is ±1 ms, it is impossible to ensure the existence of stress wave superposition for normal cutting blasting in underground mines even if detonators with the highest accuracy are used.

By increasing the spacing of two adjacent blast holes, it increases the possibility of stress wave interaction. However, the present study indicates that stress wave superposition for a 1 ms delayed blasting only occurs when the spacing is more than 4 m. For the blast holes with a diameter of 160 mm studied in this paper, the spacing of the breaking blasting is generally less than 3 m [41]. The delay scatter of several milliseconds of the pyrotechnic detonators that are normally used in practice in underground mines is unlikely to improve fragmentation via the stress wave interaction in field blasting. In addition, even if wave interaction occurs, it only marginally increases the wave intensity in a small area. This small increase in the peak tensile stress has no practical significance with respect to rock fragmentation.

The present study shows that the occurrence of stress wave superposition for blasting with a single free surface is limited to conditions that would be difficult to achieve under the existing delay accuracy of detonators. This explains why no distinct differences in rock fragmentation have been found in production blasts in underground mines, where pyrotechnic detonators with a delay scatter of several milliseconds are used. Therefore, it can be conclude that the stress wave superposition of adjacent blast holes is not an indispensable factor in the process of the formation of a common blasting crater. Based on the formation mechanism of a common blasting crater, two steps are considered to be vital: the formation of through-wall cracks between adjacent blast holes and the pushing effect of detonation gas induced by adjacent blast holes on cracked rocks in the direction of a free surface [26]. For a blast hole with fully coupled stemming in good condition, the action time for detonation gas is normally more than 10 ms [42, 43]. Thus, within the range of several milliseconds, delay times have little influence on rock fragmentation and the formation of a common blasting crater, which have also be confirmed by field blasting using detonators with big scatters in underground mines [41].

Reducing the hazardous vibrations induced by cutting blasting with a single free surface has long been a big challenge in underground mines. As blasting with a delay of several milliseconds between adjacent blast holes leads to almost the same rock fragmentation as in the case of simultaneously initiated blasting, short-delay blasting with a delay of a few milliseconds using electronic detonators can be used to control the vibration. By precisely controlling the detonation of adjacent blast holes, blasting-induced vibration could be reduced. This holds great potential for underground stopes, which are normally tens of metres away from filling stopes and mining machines. Further study regarding this problem needs to be conducted.

## Supporting information

S1 Dataset (RAR)
